# Ovarian Fibroma with Serous Cystadenoma—An Unusual Combination: A Case Report

**DOI:** 10.1155/2012/641085

**Published:** 2012-07-16

**Authors:** P. S. Jayalakshmy, Usha Poothiode, G. Krishna, P. L. Jayalakshmy

**Affiliations:** Department of Pathology, Government Medical College, Kottayam 686008, Kerala, India

## Abstract

Surface epithelial tumors account for more than 90% of ovarian tumors, of which serous tumors comprise 46%. Sex-cord stromal tumors constitute 8% of ovarian tumors, fibroma being the commonest, comprising 70% in this category. Combination of different types of tumors can occur in ovary with most common being Mucinous cystadenoma and Brenner tumour. We report a case of a very rare combination of ovarian tumour-Fibroma with Serous cystadenoma of the ovary. This combination is not mentioned in any standard textbooks or reference books of gynaecologic pathology. Extensive search of the English Literature showed only one reported case with this combination of ovarian tumors. To the best of our knowledge, this is the second case to be reported in English Literature.

## 1. Introduction

Surface epithelial stromal tumors, the most common neoplasms of the ovary, encompass five distinct subtypes, namely, serous, mucinous, endometrioid, clear cell and transitional cell along with combination of these types [1].

Tumors with serous differentiation represent 46% of all surface epithelial stromal ovarian neoplasms of which 50% are benign serous tumors [2]. These are usually cystic and tend to have thin walls and lack solid areas [3]. A few papillary excrescences may be present. Serous tumor with a solid, fibrous component is designated as serous cystadenofibroma in which both components are closely intermixed. 

Sex-cord stromal tumors account for approximately 8% of all ovarian tumors, of which fibromas account for approximately half of the cases [4]. These tumors occur at all ages with a peak in the perimenopausal age and are mostly benign. Grossly fibromas are usually solid and firm. Cystic degeneration may rarely occur in fibromas. These are pseudocysts without an epithelial lining. Fibromas may rarely have a minor component of sex cord elements, and if it is less than 10%, it is designated as fibroma with minor sex cord elements. 

Here, we report a case of a benign solid and cystic tumour of the ovary, which showed a combination of ovarian fibroma with a serous cystadenoma. This combination is very rarely reported in the literature and is not mentioned in any standard text books of Gynaecologic pathology.

## 2. Case Presentation

56-year-old patient presented with a swelling in the lower abdomen. Imaging studies showed a right ovarian mass with solid and cystic component. Suspecting a malignant ovarian neoplasm, a laparotomy and pan hysterectomy was done. No ascites or pleural effusion was present.

**Figure 1:**
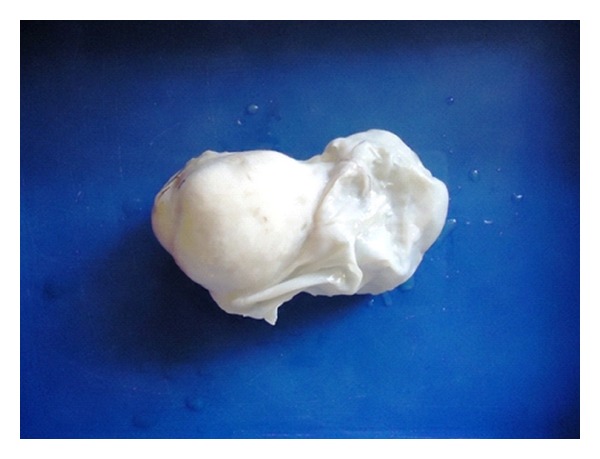
Photograph of gross specimen of right ovarian tumour: solid and cystic mass with smooth surface.

**Figure 2:**
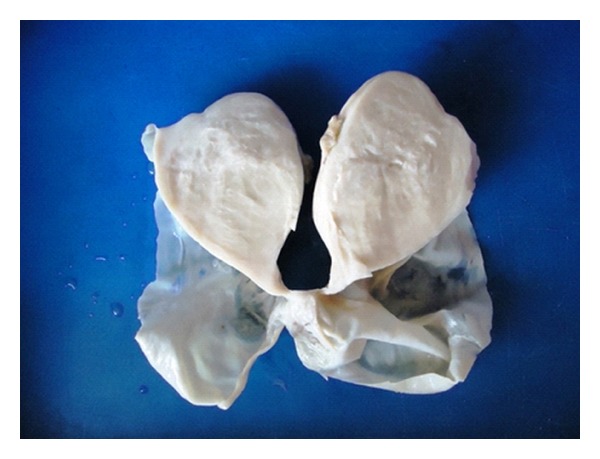
Cut section of the right ovarian tumour. Solid and cystic components are seen with clear demarcation.

Pan hysterectomy specimen was received in two pieces. One was uterus with both tubes and attached left ovary. The Right ovarian tumuor was received separately and measured 15 × 8 × 6 cms. Surface was smooth and grey white. C/S showed a solid and cystic neoplasm; the solid part measured 7 × 6 × 6 cms and was firm and fibrous. Adjacent to the border of the solid part and merging with it, there was a uniloculated cyst measuring 8 cms in greatest diameter with thin wall and containing clear fluid. Inner wall was smooth without any papillary excrescences. Uterus, other ovary, and both fallopian tubes were grossly normal (Figures 1 and 2). 

**Figure 3:**
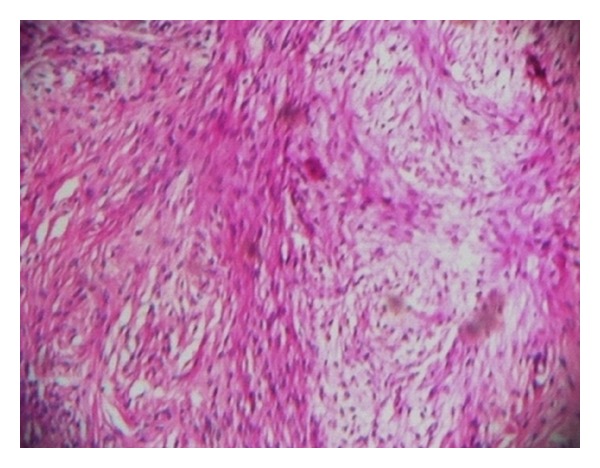
Histology of solid part: spindle cells arranged in sheets/fascicles. (H&E ×400).

**Figure 4:**
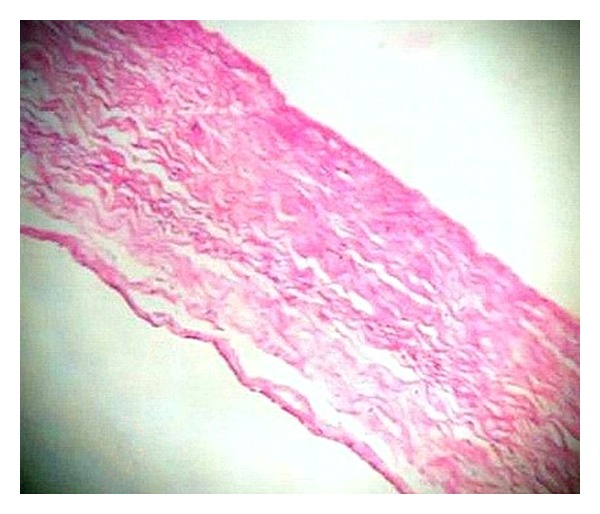
Histology of the cystic part: thin fibrocollagenous cyst wall lined by flattened/cuboidal epithelium. (H&E ×400).

**Figure 5:**
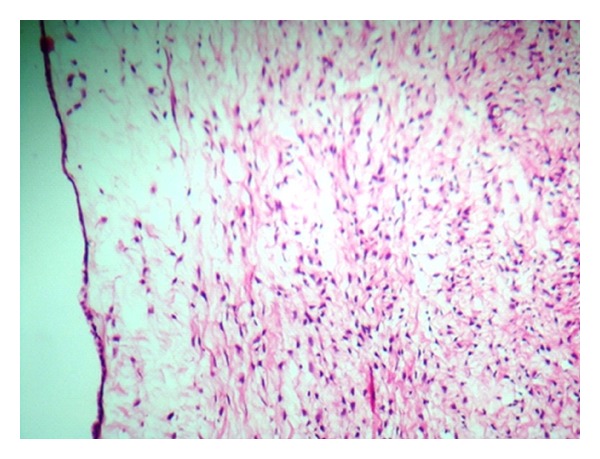
Histology of the interface between the solid and cystic region-shows lining by flattened epithelium. (H&E ×400).

Microscopy of the sections from the solid area of the ovarian tumour showed a neoplasm composed of sheets and fascicles of bland spindle cells admixed with collagen (Figure 3). The cells have scanty cytoplasm and uniform oval nuclei without atypia and mitosis. Focal areas with intercellular oedema were seen. Theca component was not present. No entrapped glandular structures were seen excluding the possibility of a serous cystadenofibroma. No transitional cell lobules were seen in the solid area even on extensive sampling ruling out a Brenner tumour. Sections from the cystic part showed a thin fibrocollagenous cyst wall lined by cuboidal to flattened epithelium (Figure 4). The lining epithelium was also seen in the interface between the solid area and the cystic part, (Figure 5) indicating that this is not a cystic degeneration of the fibroma. Hence, the diagnosis of a combined fibroma with serous cystadenoma of the ovary was made. The other ovary was normal grossly and histologically. Both fallopian tubes were unremarkable. Uterus showed proliferative endometrium and chronic cervicitis.

## 3. Discussion

Surface epithelial-stromal tumours are the commonest of ovarian tumours. Sex cord stromal tumours are less common. A combination of these tumours are very rarely encountered. The more common combinations of ovarian tumours encountered are with mucinous cystadenoma in which a combination of Brenner tumor, mature cystic teratoma, Sertoli-Leydig cell tumor, or even a serous cystadenoma may be seen [5].

Serous tumour can have a fibrous component in the subtype-serous cystadenofibroma. But, in this type, grossly cystic spaces of variable sizes are seen embedded in a markedly fibrous mass and will not be seen as clearly visible separate solid and cystic mass as in our case. Histologically, in serous cystadenofibroma, both epithelial and fibrous components will be closely intermixed. In our case, there was a clear demarcation between the fibrous solid part and serous cystic part, grossly and histologically.

Cystic change can occur in fibromas as a degenerative change, in which case, no lining epithelium will be seen in the cystic part. In the present case, cystic part was lined by flattened/cuboidal epithelium all over the wall including the interface of solid and cystic part. The lining epithelium of the cyst was flat to cuboidal rather than ciliated columnar. Although benign serous tumors are typically lined by an epithelium similar to that of the fallopian tube with ciliated and less frequently nonciliated secretory cells, cysts with flattened lining may be seen, which represent desquamation of the lining epithelium [6].

No standard text books or reference books of gynaecologic pathology mention a combination of ovarian fibroma and serous cystadenoma. Extensive search of the English literature showed only one such case report by Copland and Coleman titled “*Bilateral concomitant fibroma and serous cystadenoma of ovary*” in a 70-year-old female in an article published in 1946 [7]. To the best of our knowledge, this is the second case with this combination to be reported in English literature.

## 4. Conclusion

Clinically, because of the presence of solid and cystic component, the tumour may be mistaken as malignancy and a radical surgery may be done. Awareness of this combination may help in avoiding such mismanagements. Both components being benign, excision is curative. For the pathologists, it is important to correctly diagnose it as a combination tumour and not as cystic degeneration of a fibroma or as a serous cystadenofibroma. Extensive sampling should be done to exclude the possibility of a Brenner tumour, because the fibrous component of the Brenner tumour may overgrow the epithelial component, which makes the epithelial component undetectable if inadequately sampled. We are reporting this case for creating awareness among the pathologists and gynaecologists about the occurrence of this rare combination of ovarian tumour so that misdiagnosis and mismanagement can be avoided.
